# The Late Stages of Melanogenesis: Exploring the Chemical Facets and the Application Opportunities

**DOI:** 10.3390/ijms19061753

**Published:** 2018-06-13

**Authors:** Lucia Panzella, Atsuko Ebato, Alessandra Napolitano, Kenzo Koike

**Affiliations:** 1 Department of Chemical Sciences, University of Naples “Federico II”, I-80126 Naples, Italy; panzella@unina.it; 2 Hair Care Products Research Laboratories, Kao Corporation, Tokyo 131-8501, Japan; ebato.atsuko@kao.com

**Keywords:** melanins, chromophore, antioxidant, 5,6-dihydroxyindoles, hair dyeing, cosmetic formulations

## Abstract

In the last decade, the late stages of melanin biosynthesis involving the oxidative polymerization of 5,6-dihydroxyindole (DHI) and 5,6-dihydroxyindole-2-carboxylic acid (DHICA) have been extensively investigated. Most of the information derived from a biomimetic approach in which the oxidation of melanogenic indoles was carried out under conditions mimicking those occurring in the biological environment. Characterization of the early oligomers allowed for drawing a structural picture of DHI and DHICA melanins, providing also an interpretative basis for the different properties exhibited by these pigments, e.g., the chromophore and the antioxidant ability. The improved knowledge has opened new perspectives toward the exploitation of the unique chemistry of melanins and its precursors in cosmetic and health care applications. A noticeable example is the development of an innovative hair dyeing system that is based on the marked ease of DHI to give rise to black melanin on air oxidation under slightly alkaline conditions. The advantage of this method for a step-wise coverage of gray hair with a natural shade pigmentation on repeated treatment with a DHI-based formulation with respect to traditional dyes is presented. A variant of DHICA melanin combining solubility in water-miscible organic solvents, an intense chromophore in the UltraViolet-A UV-A region, and a marked antioxidant potency was evaluated as an ingredient for cosmetic formulations.

## 1. Melanin Pigmentation and Melanocytes

Skin pigmentation in humans is mainly due to the formation, quantity, type, and distribution of melanins in the epidermis [1,2]. The resulting pigmentation is also determined by the phenomena of light reflection and diffusion, and by the presence of pigments of different kind, occasionally present in the skin. Apart from the hemoglobin and the biliary pigments, melanins are the only pigments to be biosynthesized in humans. Two main groups of intracellular nitrogenous pigments are found in humans, the black-to-dark brown eumelanins and the lighter, yellowish-to-brown, sulfur containing pheomelanins [3,4].

Beside skin, melanins determine the color of human hair [5,6,7,8]. This depends on the quantity, quality, and distribution of melanins, but also on the ratio of eumelanins to pheomelanins [9,10,11]. Caucasian people have a variety of hair colors from light blond to black. On the other hand, Mongoloids, such as Asian people, including Chinese and Japanese, have only black hair exhibiting high levels of eumelanin [12]. Microscopic observation of melanin pigment in the cross section of Japanese hair shaft showed that the distribution is not regular or even, and that it tends to be more in the outside of the cortex, less in the inside [12].

Melanins are also found in the eyes, in the irides, whose color is determined again by the pigment levels and the eumelanin/pheomelanin ratio, and in the uveal tract [13], in the inner ear [14], in the leptomeninges of central nervous system, and in some mucous membranes [3].

Melanins are produced in melanocytes, cells that are capable of synthesizing the enzyme tyrosinase, which, when incorporated into specialized organelles called melanosomes, promotes a series of events leading to the synthesis and the accumulation of the pigments [1]. During the embryonic development, they migrate from the neural crest in the form of melanoblasts to reach the skin at the dermal-epidermal junction, the choroid of the eye, and the hair bulbs where they differentiate into typical melanocytes. Differently, melanocytes of the retina derive from the outer wall of the optic vesicle. The structure and the shape of melanosomes change during their development [15], and the matured, highly melanized, and electron-dense melanosomes are transferred from melanocytes to neighboring cells, keratinocytes [2,16]. Melanosomes predominantly synthesizing eumelanin, eumelanosomes, usually appear to be ellipsoidal in shape (approx. 1 × 2 μm), whereas pheomelanosomes appear to be spherical [6]. These two types also differ in the morphology of the membrane on the surface and inside cavities, as documented by three-dimensional images [17,18].

## 2. Melanogenesis with Focus on the Late Stages

**Figure 1:**
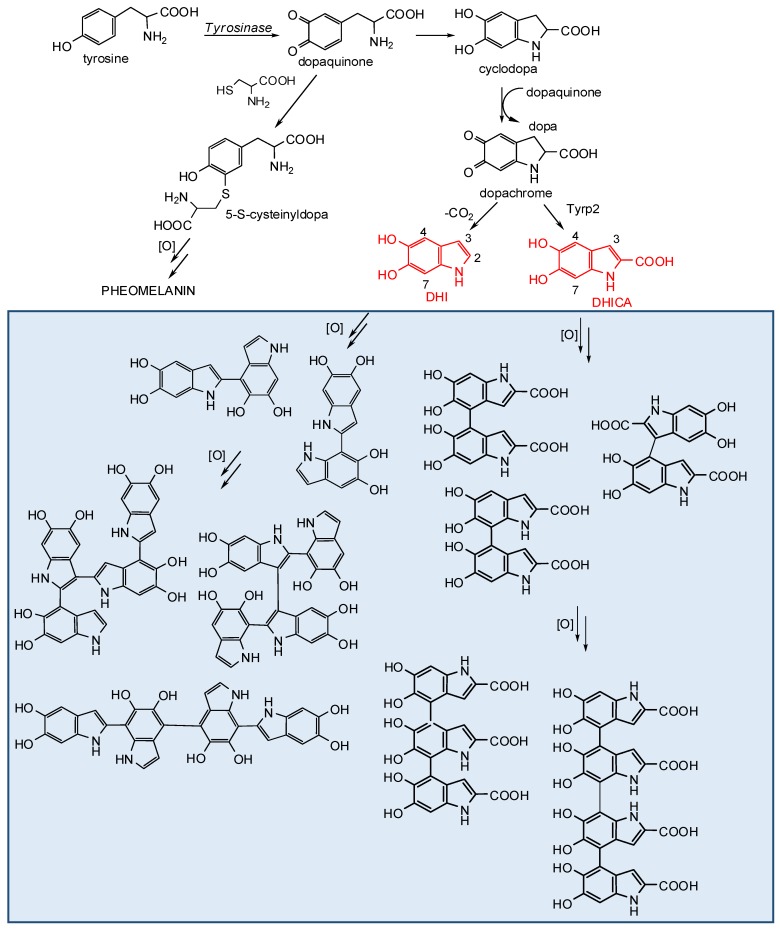
Schematic overview of melanogenesis. The late stages beyond formation of 5,6-dihydroxyindoles DHI and DHICA (in red) are highlighted and the mode of oxidative coupling exemplified by the structures of the main oligomers so far identified [4,33,34].

Eumelanins and pheomelanins are both biosynthesized by tyrosinase catalyzed oxidation of the amino acid l-tyrosine leading to dopaquinone (Figure 1). This latter is highly reactive and undergoes intramolecular cyclization yielding to cyclodopa (or leucodopachrome). Dopachrome, which is an orange-red intermediate, is then generated by a redox exchange between cyclodopa and dopaquinone together with dopa. This process therefore gives rise to dopa that is formed during melanogenesis (Figure 1). Rearrangement of dopachrome proceeds mostly with decarboxylation, leading to 5,6-dihydroxyindole (DHI) together with 5,6-dihydroxyindole-2-carboxylic acid (DHICA) to a lesser extent [19,20,21].

As to pheomelanin biosynthesis, the reaction of cysteine with dopaquinone gives rise exclusively to cysteinyldopas adducts, with 5-*S*-cysteinyldopa as the major isomer [21,22]. Oxidation of the thiol adducts leads to benzothiazine intermediates that are then converted to the pigment [23].

Beside tyrosinase, another tyrosinase-related protein intervenes in eumelanogenesis, that is dopachrome tautomerase (Dct), or tyrosinase-related protein-2 (Tyrp2), which catalyzes the tautomerization of dopachrome to DHICA [24,25].

The stages of melanogenesis beyond the formation of the monomer 5,6-dihydroxyindoles leading to the eumelanin pigments have been extensively investigated. Several issues are still controversial though it is generally agreed that these involve an oxidative polymerization process. Firstly the factors responsible for the oxidation of 5,6-dihydroxyindoles in vivo are far to be defined. Mammalian tyrosinase has been reported to be able to promote oxidative polymerization of DHI [26]. A pulse radiolysis investigation provided evidence that DHI can be effectively oxidized by dopaquinone [27]. In mice, another tyrosinase-related protein, Tyrp1, can oxidize DHICA [28,29]. On the other hand, human Tyrp1 is apparently unable to catalyze this process [30], but it can oxidize tyrosine, dopa, and DHI [21]. The quantity and quality of eumelanins that are produced in terms of the ratio of DHI to DHICA-derived units and the degree of polymerization is therefore greatly affected by the activities of these tyrosinase-related proteins [31].

The mechanism of the oxidative conversion of melanogenic indoles to eumelanins have been the subject of intensive research work. Most of the information that is presently available derives from a biomimetic approach in which the oxidation of the indoles is carried out under conditions that are mimicking those occurring in the biological environment. Either indoles are converted to black insoluble pigments resembling natural eumelanins following exposure to oxidizing enzymes, UV radiation, chemical oxidants, or even on standing in air at neutral physiological pHs, although with a different kinetics [32].

Oxidation of DHI proceeds rapidly, leading to a series of dimers and trimers, in which the indole units are linked through 2,4′- and 2,7′-bondings [33,34,35] (Figure 1). Tetramers generated by oxidative coupling of dimers were found to exhibit other types of interring bonds, e.g., 2,3′-, 4,4′-, and 7,7′-bonds, depending on the starting dimer [36,37,38,39]. As a result of these studies a mechanism of polymerization of DHI can be drawn in which monomer units couple through the 2- and 4- or 7-positions. Yet, when oligomer coupling becomes predominant, other modes of bonding may prevail, leading to a bending of the growing oligomer chain. The structural diversity that is generated during eumelanin biosynthesis is expectedly high, and this would account for the marked heterogeneity of the pigment.

Beside linear oligomers, cyclic oligomer components featuring a porphyrin-like structure have been originally proposed on a theoretical basis by Kaxiras in 2006 [40]. In this structural model of eumelanin, porphyrin-type building blocks that were derived from the oxidative cyclization of DHI tetramers built via 2,7′-coupling would form highly planar structures that are capable of strong π-stacking interactions [40,41]. Following those studies, a number of other research works have addressed this issue, either by computational analysis exploring the relative stability of the various oxidation states, their aromatic character, and tautomeric equilibria [42,43] or by direct experimental investigation of synthetic DHI melanins by mass spectrometric techniques, like Matrix Assisted Laser Desorption Ionization Mass Spectrometry (MALDIMS) [44,45,46,47]. Though chemical consideration would argue against porphyrin formation that seems unlikely under a statistical view point as it requires four sequential 2,7′-coupling steps and a final cyclization unless any template system is involved, this fascinating model has not definitely been abandoned.

In the case of DHICA, the electron-withdrawing nature of the 2-carboxylic acid group decreases the nucleophilic reactivity of the pyrrole moiety and the polymerization coupling is mainly directed towards the 4,4′-, 4,7′-, and 7,7′-bonding patterns, whereas the 3-position is involved to a minor extent only (Figure 1) [33,38,39].

## 3. Structure-Properties Relationship of Melanin Pigments

One of the most peculiar features of eumelanins are their optical properties, and particularly the black color. In the past decade, it was suggested that eumelanin broadband absorption properties could be explained in terms of the chemical disorder model [48,49], which envisages the overlap of a series of chromophores that are spanning the entire UV-visible range.

A major obstacle to the investigation of the eumelanin chromophore is represented by the unsolubility of the final pigment. A water-soluble eumelanin model polymer was therefore produced from a glycosilated DHI derivative and the investigation of this model pigment provided evidence for the first time that the black chromophore reflects the coexistence of oxidized and reduced moieties within oligomer/polymer scaffolds [50].

A further stepforward stemmed from the discovery that addition of some percent of poly(vinyl alcohol) (PVA) in the aqueous buffer in which the pigments are generated can prevent the precipitation of growing eumelanin polymers. The use of this additive therefore allowed further investigation of eumelanin chromophore disentangled from scattering effects [51,52].

**Figure 2:**
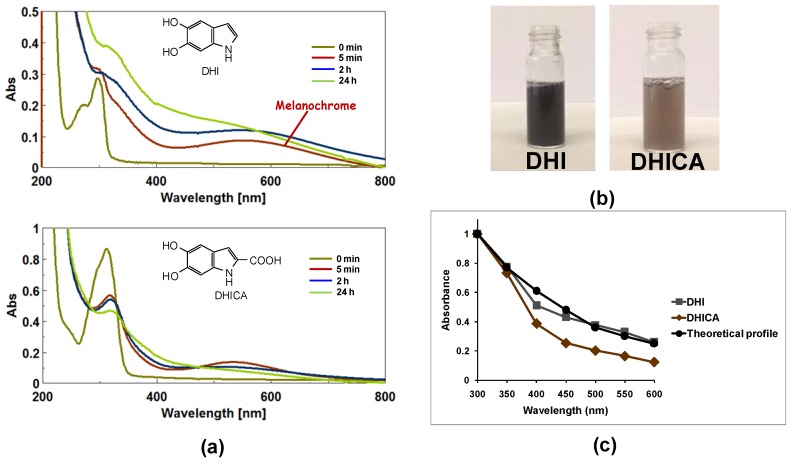
Melanin chromophore from 5,6-dihydroxyindole (DHI) or 5,6-dihydroxyindole-2-carboxylic acid (DHICA) oxidation. (**a**) Time course of DHI (**top**) or DHICA (**bottom**) aerial oxidation; (**b**) Final appearance of the mixture of DHI or DHICA after 24 h oxidation; and, (**c**) Relative absorbance of the melanins from the indole compounds over the UV-visible region in the presence of 1% poly(vinyl alcohol) (PVA) against the theoretical monotonic profile [53].

In a recent study, advances in the understanding of the process underlying formation and evolution of eumelanin chromophore stemmed from a comparative investigation of the dynamics of the oxidation of DHI and DHICA in aqueous buffers under different conditions [53]. Generation of a broad visible chromophore around 560 nm, termed melanochrome, was observed in the first minutes of DHI oxidation, while on standing the solution slowly darkened both in air and in an argon atmosphere due to broadening of the absorption band accompanied by extensive precipitation at 24 h. Based on these and other results from mechanistic experiments, it was possible to draw a relatively simple and well defined picture of eumelanin chromophore buildup involving three phases: phase I, i.e., the generation of intensely absorbing melanochrome, which is fast, depends on the availability of the oxidant, and it reflects mainly intrinsically-defined chromophores, e.g., oligomer species at various oxidation levels; phase II, a band broadening process leading to solution darkening, which is slow and proceeds in an oxygen-independent manner, as is consistent with intermolecular chromophore perturbations accounting for extrinsically determined absorptions; and phase III, the onset of scattering due to eumelanin precipitation (Figure 2a, top panel) [53].

A markedly different spectrophotometric course was observed in the case of DHICA (Figure 2a, bottom panel). In this latter case, a deep violet chromophore broadly centered between 500 to 550 nm was generated, accompanied by a well-defined UV absorbing band around 330 nm, slightly shifted relative to the monomer maximum. Visual inspection of the reaction mixtures from DHICA again revealed the important role of aggregation in color development corresponding to the broadening of the visible absorption maximum. The most noticeable difference of the oxidation of DHICA with respect to DHI oxidation was the lighter coloration of the final melanin (Figure 2b) [53].

Shown in Figure 2c are the shapes of the final melanin curves from DHI and DHICA, as obtained by normalizing absorption at fixed visible wavelengths against the UV band at 300 nm. It appears that DHI melanin more closely matches the theoretical monotonic profile with respect to DHICA melanin. Such differences may be well interpreted in the light of the structural features of DHI and DHICA oligomers. Planar conformations can be envisaged for DHI oligomers, whereas in DHICA polymers, the presence of biphenyl-type bonds results in non-planar, partly linear backbones in which rotation around the interunit bonds is partly hindered (atropisomerism) [38,39]. Such a deviation from coplanarity in the oligomers/polymers from DHICA is further supported by the repulsion of negatively charged carboxylate groups. As a result, the twisted backbones are not amenable to give rise to π-stacked supramolecular aggregates, differently from the planar oligomeric scaffolds derived from DHI. So, strong absorption in the visible region is obtained for the largely planar species that are generated from the oxidation of DHI dimers, whereas no significant visible chromophore above 400 nm is produced by DHICA oligomers, for which inter-unit dihedral angles of ca. 49° with localized *o*-quinone moieties and the interruption of inter-unit π-electron delocalization has been predicted [54,55].

All of these data contributed to formulate a new, more detailed interpretation of the black color for synthetic DHI and DHICA eumelanins. Intrinsic effects relating to efficient π-electron delocalization within planar oligomeric scaffolds is the main determinant of DHI-melanin chromophore, while the black chromophore of DHICA melanin is mainly extrinsic in character deriving from aggregation-dependent intermolecular perturbations of the π-electron systems.

These structural pictures of DHI and DHICA melanins provides a useful interpretative basis also for the different reactivity observed for DHI and DHICA melanins. DHICA-melanin dose-dependently acts as a potent OH radical scavenger in the Fenton reaction, whereas, in the same range of concentrations, DHI-melanin is rather a pro-oxidant capable of generating reactive oxygen species [56]. In addition, DHICA melanin is much more effective as H-donor and NO scavenger with respect to both DHI and dopa melanin [57].

Interestingly enough, DHICA monomer proved as well a highly efficient OH radical scavenger and it performed very well as a ferric reducing antioxidant [25]. Though a direct comparison of the potency of DHICA and its melanin is not possible because of the much different solubility properties and variable conditions adopted in the relevant studies, it may be concluded that polymerization does not change substantially the antioxidant character of the indole in agreement with the view that for this pigment the inter-unit π-electron delocalization of the indole units provides only a modest contribution in the polymer backbone.

## 4. Exploiting the Uniqueness of Melanin Chemistry

The peculiar reactivity of melanogenic intermediates that is finely tuned by several factors including the presence of carboxyl groups as highlighted in a recent review article [24], but also the mode of coupling of the indole units, as indicated by the much different chromophores that developed on the oxidation of DHI and DHICA and their oligomers [53,58] have inspired the development of several applications in the field of health care and cosmetics that may prove of considerable interest. In addition, the marked antioxidant character and the reactive oxygen species scavenging ability of the eumelanin pigments has been considered as a valuable model for the design of innovative all-natural cosmetic ingredients.

### 4.1. Hair Dyeing with Melanin Precursors

The ease with which intermediates in melanogenesis undergo air oxidation without enzymes or other catalysts giving rise to intensely colored brown to black solutions makes them optimal candidates for hair dyeing, particularly for the coverage of gray and white hairs.

#### 4.1.1. Gray Hair and Hair Dyes

A main biological role of human hair is the protection from sunlight and heath, as exemplified by hair of African people, in which the unique kinky (curly) shape and the black color are essential for playing such a role [5]. Studies on curvature of human scalp hair and crimp of wool have shown that the bilateral structure (*ortho*- and *para*-cortex) is one reason for hair curling [59,60]. Comparison of the morphological and ultrastructural features of naturally straight and curved human scalp hairs showed that the distribution of the cortical cells were different: symmetric in straight hair, and asymmetric in curved hair. De Galvez and Dario recently stressed the critical photoprotective role that is played by hair based on epidemiological evidence that individuals with balding or thinning hair are at greater risk of skin cancer and introduced a hair ultraviolet protection factor (HUPF) for a quantitative evaluation [61,62]. It appears that hair provides a barrier against both UVB and UVA radiation as a function of hair density, thickness and mostly the presence of melanin. This is a topic still not fully explored, but is of great interest also in light of the much different response of skin to UVB, UVA and simulated solar radiation in terms of erythema, DNA damage, apoptosis, and tanning [63,64].

These several types of cutaneous responses strongly depend on the wavelength of the radiation [65]. UVA radiation causes immediate pigment darkening and persistent pigment darkening, both of which are the result of the oxidative polymerization of existing melanogenic precursors by the action of reactive oxygen species [66,67]. On the other hand, UVB radiation causes the delayed tanning reaction that is developed over several days [68], as it involves production of melanin as a result of the increased activity of tyrosinase, although other factors, such as the redistribution and the particle size of melanosomes, are important as well [69].

A progressive depletion of melanocyte stem cells in hair follicles has been identified as the main cause of hair graying, which leads ultimately to a failure in the renewal of the hair pigmentation unit at the telogen to anagen transition during hair cycle, and ultimately to the growth of white hair [70].

Since the ancient times, gray hair coverage has been performed by various methods employing chemicals, metals, and also natural products [5,71,72,73]. As a remarkable example, the Japanese “tale of the Heike”, which was written in 14th century, tells about an old soldier dyeing his hairs before entering battle.

Hair dyes based on the combination of oxidizable color-developing compounds and hydrogen peroxide as the oxidant are used worldwide because of their effectiveness and convenience, though this technology still has some problems, such as complicated handling, hair damages, color fading (stability or durability), and safety [74,75,76,77,78,79].

Practically, the hair damages that are caused by hair dyes are related to the behaviors of consumers, not only the frequency of hair dyeing, but also shampoo procedures and prolonged dryer usage [12,80,81]. As hair growth rate is approximately 1 cm length per month [5], physical and chemical damages, both inside and outside the hair dramatically accumulate; for example, a 25 cm length hair has a two-year history of hair care behavior. By microscopic observation of the hair from scalp to tip the progression of the damage can be evaluated. Originally, the hair surface is hydrophobic, because it is covered with 18-methyl eicosanoic acid (18-MEA), a main component of the outer layer of the epicuticle of human hair [80]. Its removal caused by oxidative dyes makes the surface more hydrophilic and initiates the damage: flow-out of proteins and lipids, cuticle layer reduction, and the appearance of cavities inside the cortex and cuticle. Therefore, new safe and reliable technologies for hair dyes that avoid hydrogen peroxide and oxidative dyes are urgently needed.

Might melanin be restored in gray hairs this would result in a revitalization of the hair turning the color to the natural black. This straightforward approach however is not feasible as melanins are very large molecules that cannot penetrate into gray hair. So, they cannot be used as dyestuffs for hair coloring.

A valuable alternative was the development of melanin from its precursors inside the hair. Along this line an interesting innovative approach was developed in recent study reporting the use of polydopamine, which is a eumelanin type polymer derived from oxidation of dopamine, for the dyeing of gray Asian hairs. Application of dopamine to hair in the presence of ferrous ions allowed in a reasonable time of 1 h to get black colorations that proved to be fairly stable to washings and non-toxic in model animals. Three kinds of deposition mechanisms i.e., innate binding ability of polydopamine, metal-assisted self-assembly of polydopamine, and metal-related bridging between keratin surface and polydopamine were proposed [82].

#### 4.1.2. Melanin Precursors and Their Selection for Hair Dyes

**Figure 3:**
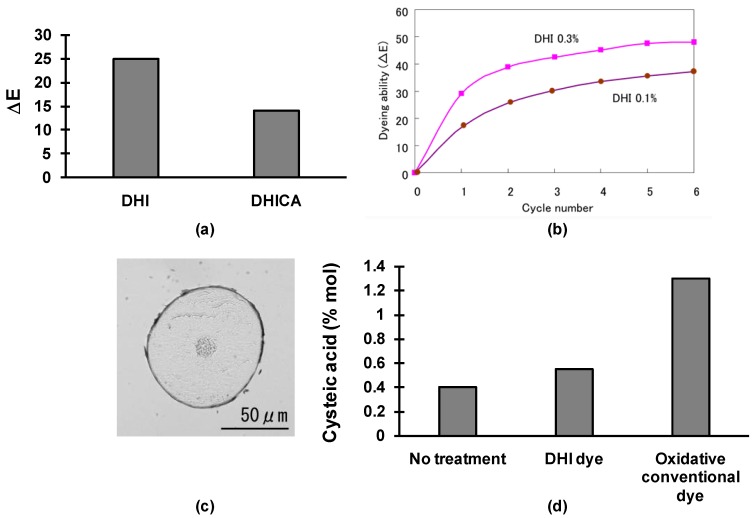
Development of DHI-based hair dye. (**a**) Comparison of the dyeing performance of melanin precursors. Dyeing level (∆E) represents the difference of the color measured with a CR-400 (Minolta, Japan) color photometer before and after three times treatment of 1 g of Japanese gray hair tresses treated for 5 min with 1 g of a solution containing 3% ammonia, and 0.1% melanin precursor at room temperature; (**b**) Dyeing ability of the DHI hair dye on gray hair. The hair dye contained 0.1 or 0.3% melanin precursor (DHI), 1% alkaline agents (2-aminoethanol), 10% organic solvents (ethanol and butylene glycol), 0.5% a thickener (acrylates/C10-30 alkyl acrylate crosspolymer), 0.5% silicones (dimethicones and amodimethicones), 1.5% nonionic surfactant (Polyoxyethylene (9) dodecyl ether), 0.5% antioxidants (ascorbic acid and sodium sulfite). 1 g of gray hair tresses were treated with 1 g of a dyeing formulation, left for 5 min at room temperature, shampooed twice, and dried with a hair dryer. This cycle was repeated up to six times. Dyeing ability (∆E) defined as in (**a**); (**c**) Cross section microgram of the gray hair after eight treatments; and, (**d**) Levels of cysteic of hair samples treated with DHI based dye (five treatments) or with conventional hair dyes.

With the aim of selecting the best candidate for hair dyeing formulation, different melanin precursors, including DHI, DHICA, tyrosine, and dopa were preliminary evaluated for their ability to give rise to melanin in air under alkaline condition (3% ammonium hydroxide solution) at 0.1% concentration over time. DHI and to a lesser extent DHICA gave rise to the rapid development of an intense color, whereas tyrosine and dopa did not. On this basis the selected compounds were assayed for their ability of dyeing gray hairs. DHI performed much better, as shown in Figure 3a, and was therefore further investigated as a promising candidate for the development of an innovative hair dye.

The advantages of using DHI as hair dyeing agent with respect to conventional oxidative hair dyes may be summarized, as follows:

(1) Sustainable product: this dyestuff is a natural origin and sustainable material.
(2) Genuine natural shade results: DHI produces original color of melanin.
(3) Hydrogen peroxide-free formulation: it means no need to mix (easiness to use), and less damage on hair.

In order to develop an efficient and industrial production of DHI for hair dyes, a biotechnological approach was developed (Kao Corporation. Production process for indoles and indolins. JP-Patent 4653333, 30 March 2001).

##### DHI Manufacturing Process

Japanese sake is a traditional alcoholic drink, which is sometimes called rice wine, which is obtained by a long fermentation process similarly to wine. However, while in the case of wine a single fermentation step, i.e., yeasts converting sugar in grape to alcohol, is required, different stages are needed to obtain sake. Firstly starch in rice is converted to sugar by the fungus *Aspergillus oryzae*, and then the sugar is converted to alcohol by yeasts, like *Saccharomyces cerevisiae*. Thus the sequential action of the different microorganisms produces the desired transformation. Based on genomic and enzymological examinations, it has been reported [83] that *Aspergillus oryzae* has a unique tyrosinase, *melB* which is expressed only in solid cultivation and it led to the formation of brown products. Later, this finding proved helpful not only for sake brewing, but also for DHI production [84].

In the early stages of the production trials, it was very difficult to accumulate efficiently DHI by using *Aspergillus* tyrosinase. The yield was very low because oxygen stimulated both DHI production and melanin formation from DHI. The initial reaction, the conversion of dopa to dopachrome by tyrosinase, was accelerated by optimization of the reaction conditions, such as increasing the enzyme activity, air supply, and pH management. After effective conversion of dopa to dopachrome (yield > 60%), further conversion to DHI was slowed down and controlled by oxygen purging and pH tuning. The product and catalyst were then separated from the reaction mixture by filtration, and the concentration of DHI was adjusted for use in dyes. Formulation of the dye was then accurately developed.

Formulations containing 0.1% or 0.3% DHI were tested for the dyeing ability by repeated step-wise treatments, as shown in Figure 3b. Efficient coverage of gray hair was obtained with 0.3% DHI in the formulation that reached a color difference value ∆E) of 40.

The new hair dye that was based on the use of DHI as a key material was eventually launched on the market (Gekkeikan Sake Company; Kao Corporation. Hair dye composition. JP-Patent 4955920, 4 December 2004, and Gekkeikan Sake Company; Kao Corporation. Hair dye composition. JP-Patent 4395436, 4 December 2004).

##### Dyeing Mechanism and Advantages of the Use of the DHI-Based Dye

After eight treatments with 0.3%DHI formulations the dyed part of the hair where melanin accumulated proved to be only the cuticle (TEM and light microscopy analysis, Figure 3c), which is the outer part of the hair, whereas natural melanin is present in the cortex part of the hair [5,12], suggesting a low durability of the color. However, in the durability test, for example after 20 washings, the performance of DHI hair dye was very good. The structural features of the final melanin generated from DHI was investigated by MALDI-TOF mass spectrometry. It was found that polymerization of DHI proceeded promptly, as assessed by monitoring DHI decay in the formulation on air exposure. The molecular weight of melanin formed from DHI ranged from 1000 to 5000 Da, with an average value of about 2500 Da. This means that polymers containing more than 10 units of DHI were obtained. The significant extent of polymerization of DHI could account for the high durability of the dye. For comparison, conventional oxidative dyes, such as *p*-phenylenediamine, polymerize to give oligomers, mostly dimers or trimers, with a molecular weight lower than 500 Da.

Based on all these observations, the dyeing mechanism may be described as follows: at first, when the hair dye is applied to gray hair, DHI penetrates into the cuticle of the hair, and converts there to melanin by air oxidation in a few minutes. However, gray hair is only slightly dyed by the melanin formed, but by step-wise use, that is, repeating the cycle of treatments, the melanin accumulates in the cuticle, and the hair color becomes gradually darker and darker. After several applications, gray hair turns to naturally dark shades.

For the assessment of hair damage, the levels of cysteic acid arising from oxidation of disulfide bridges of cystine residues of hair keratin and decrease of 18-MEA on the surface were measured by HPLC and GC, respectively. In either case, the results that were obtained with hair treated five times were almost equal to untreated hair, indicating a very low level of damage that is associated to the dyeing treatment (Figure 3d). Staining of the skin, which represents a major limitation of direct dyes, was also quite low. Overall these results indicate remarkable advantages of the use of the DHI-based dyeing system over the conventional technologies.

Evaluation of the performance by panelists indicated that the main strengths of this new system was the natural origin of the dyestuff, the gradual change of the color of hair from gray to natural black on repeated treatment, and the ease to run the hair treatment.

In conclusion, this application opens interesting perspectives in the use of melanin precursors for the development of all natural hair dyes. It is expected that a variety of hair hues can be achieved by proper manipulation of the process e.g., by use of precursors with different melanization kinetics or by the use of additives that can affect the structural properties and hence the chromophore of the final melanin pigments.

### 4.2. Eumelanin Based Cosmetic Ingredients

Consideration of the peculiar chromophore of DHICA melanins, covering the UVA region that is generally agreed as the most dangerous portion of the solar spectrum that is responsible for damages to cellular components associated to sun exposure [85] would suggest their exploitation as ingredients in dermocosmetic formulations with photoprotective action. The superior antioxidant activity of DHICA melanins [56,57] would strengthen this action ensuring protection against oxidative stress processes that are triggered by the exposure to solar radiation. Limitations to the use of these materials however derive from their low solubility in hydroalcoholic or more lipophilic media that are usually employed in cosmetic formulations and the ease to undergo degradation e.g., by photooxidation under UVA as a result of quenching of singlet oxygen with loss of their properties, as recently reported [86].

A variant of DHICA melanin that was obtained from the polymerization of the methyl ester of DHICA (MeDHICA) was recently developed [87]. MeDHICA was prepared by a procedure that is based on a biomimetic reaction involving oxidative cyclization of the methyl ester of dopa, and then subjected to oxidative polymerization in air under slightly alkaline aqueous buffer.

**Figure 4:**
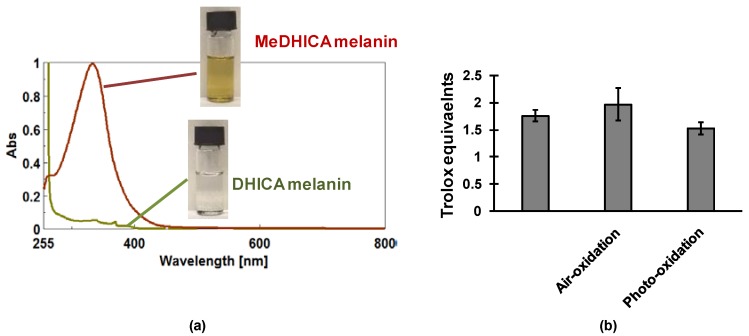
(**a**) Absorption properties and color of a solution of methyl ester of DHICA (MeDHICA) melanin in DMSO. For comparison a suspension of DHICA melanin under the same conditions is shown; (**b**) Ferric reducing antioxidant power (FRAP) assay of MeDHICA melanin before and after exposure to air over one week or photoxidation under solar simulator light for 3 h.

The pigment thus obtained exhibited an intense and broad chromophore centred at 330 nm with almost no absorption in the visible region. Most notably MeDHICA melanin exhibited a fairly good solubility in different water miscible organic solvents, like dimethylsulfoxide (DMSO) or acetonitrile reaching concentrations up to 1.5 mM based on the starting monomer, but more limited in alcohols and glycerol. On the other hand, under these conditions, DHICA melanins proved almost completely unsoluble (Figure 4a).

This material was shown to consist of a mixture of intact DHICA methyl ester oligomers up to the heptamer, and shared the structural features that were described for DHICA melanins, including the free radical character, indicating the presence of oxidized and reduced indole moieties that are capable of giving rise to semiquinone radicals by comproportionation equilibria. The antioxidant activity as H-donor and ferric ion reductant ability of this melanin was remarkable also when compared with the reference compound Trolox. Most interestingly, the material proved stable to air over weeks or following exposure to the light of a solar simulator over hours with no changes in the chromophoric features. Also, the antioxidant power as exemplified by the ferric ion reduction power was almost unaltered following prolonged exposure to air or photoirradiation (Figure 4b).

## 5. Conclusions

Based on the overview that is offered by this presentation, it can be concluded that the chemistry of the late stages of melanogenesis is intriguing and exhibits several peculiar aspects, first of all the great differences of reactivity to oxidation and kinetics of conversion to melanins shown by the two melanogenic indoles DHI and DHICA, and secondly, the structural diversity of the oligomers that is dictated mostly by the electronic effects of the carboxyl group, but does not depend on the oxidizing conditions adopted. This also warrants that model studies mimicking the oxidative conversion of each of the indoles to melanins may provide useful information on the structural features of natural pigments, though these latter comprise units that were derived from either component to a different still not defined extent. Based on these investigations, we may now correlate the much different properties of these materials to their structural diversity that is an extended π-electron delocalization within largely planar scaffolds for DHI melanins and non-planar, partly linear backbones exhibiting hindered rotation at the interunit bonds for DHICA melanins.

The improved knowledge of the melanization process and the structural features of the final pigments has stimulated the exploration of possible applications relying on the rapid melanization of DHI with generation of deeply colored black melanins as well as on the powerful antioxidant ability of DHICA melanins and their intense chromophore in the UVA region. Examples of promising applications has been provided and it can be expected that further characterization of the chemical facets of these processes may guide future work that is aimed at manipulating these compounds to ameliorate their properties and performance for cosmetic and health care purposes.

## 6. Patents

1. Kao Corporation. Production process for indoles and indolins. JP-Patent 4653333, 30 March 2001.
2. Gekkeikan Sake Company; Kao Corporation. Hair dye composition. JP-Patent 4955920, 4 December 2004.
3. Gekkeikan Sake Company; Kao Corporation. Hair dye composition. JP-Patent 4395436, 4 December 2004.
4. Kao Corporation. Production process for indoles and indolins. JP-Patent 5507786, 7 June 2006.
5. Kao Corporation. Production process for indoles and indolins. JP-Patent 5043369, 7 June 2006.
6. Kao Corporation. Production process for indoles and indolins. JP-Patent 5043368, 7 June 2006.

## Abbreviations

18-MEA	18-methyl eicosanoic acid
Dct	dopachrome tautomerase
DHI	5,6-dihydroxyindole
DHICA	5,6-dihydroxyindole-2-carboxyl acid
HUPF	hair ultraviolet protection factor
MALDI MS	Matrix Assisted Laser Desorption Ionization Mass Spectrometry
PVA	Polyvinyl alcohol
Tyrp2	tyrosinase-related protein-2
UV-A	UltraViolet-A
